# Lipopolysaccharide Does Not Alter Small Airway Reactivity in Mouse Lung Slices

**DOI:** 10.1371/journal.pone.0122069

**Published:** 2015-03-30

**Authors:** Chantal Donovan, Simon G. Royce, Ross Vlahos, Jane E. Bourke

**Affiliations:** 1 Lung Health Research Centre, Department of Pharmacology and Therapeutics, University of Melbourne, Parkville, Victoria, Australia; 2 Department of Pharmacology, Monash University, Clayton, Victoria, Australia; 3 School of Health Sciences, Health Innovations Research Institute, RMIT University Bundoora, Victoria, Australia; Chinese Academy of Sciences, CHINA

## Abstract

The bacterial endotoxin, lipopolysaccharide (LPS) has been associated with occupational airway diseases with asthma-like symptoms and in acute exacerbations of COPD. The direct and indirect effects of LPS on small airway reactivity have not been fully elucidated. We tested the hypothesis that both *in vitro* and *in vivo* LPS treatment would increase contraction and impair relaxation of mouse small airways. Lung slices were prepared from naïve Balb/C mice and cultured in the absence or presence of LPS (10 μg/ml) for up to 48 h for measurement of TNFα levels in conditioned media. Alternatively, mice were challenged with PBS or LPS *in vivo* once a day for 4 days for preparation of lung slices or for harvest of lungs for Q-PCR analysis of gene expression of pro-inflammatory cytokines and receptors involved in airway contraction. Reactivity of small airways to contractile agonists, methacholine and serotonin, and bronchodilator agents, salbutamol, isoprenaline and rosiglitazone, were assessed using phase-contrast microscopy. *In vitro* LPS treatment of slices increased TNFα release 6-fold but did not alter contraction or relaxation to any agonists tested. *In vivo* LPS treatment increased lung gene expression of TNFα, IL-1β and ryanodine receptor isoform 2 more than 5-fold. However there were no changes in reactivity in lung slices from these mice, even when also incubated with LPS *ex vivo*. Despite evidence of LPS-induced inflammation, neither airway hyperresponsiveness or impaired dilator reactivity were evident. The increase in ryanodine receptor isoform 2, known to regulate calcium signaling in vascular smooth muscle, warrants investigation. Since LPS failed to elicit changes in small airway reactivity in mouse lung slices following *in vitro* or *in vivo* treatment, alternative approaches are required to define the potential contribution of this endotoxin to altered small airway reactivity in human lung diseases.

## Introduction

Inflammation in the distal lung plays a crucial role in many diseases including asthma and chronic obstructive pulmonary disease (COPD). The bacterial endotoxin, lipopolysaccharide (LPS) induces inflammation and has been associated with occupational airway diseases with asthma-like symptoms [1] and with acute exacerbations of COPD [2]. LPS is commonly present within house dust, which contains a common asthma allergen, and a direct correlation between levels of LPS in house dust and asthma severity has also been demonstrated [3]. Furthermore, increased bacterial load in the airways of asthmatic patients impairs steroid responsiveness and bronchodilator responses to β-adrenoceptor agonists [4]. Despite the increasing interest in contribution of small airways in the distal lung as sites of increased inflammation and altered reactivity in asthma and COPD [5], the effects of LPS on their sensitivity to constrictor and dilator agents remains to be characterized.

LPS binds to and activates toll-like receptor 4 (TLR4) [6] to induce signaling via conventional pro-inflammatory pathways such as: myeloid differentiation factor (MyD)88/NFκB; phosphoinositide 3-kinase (PI3K)/Akt; and MAP/ERK/JNK/p38. Signaling through these pathways leads an activation of Th2 cells involved in the adaptive immune system and subsequent release of cytokines, such as IL-6, TNFα and IL-1β. In addition to these conventional pathways, LPS has been shown to increase anti-inflammatory cytokines such as IL-10 and IL-22 [7], and activation of the arachidonic acid pathway leading to increased metabolites such as PGE_2_ [8]. In the airways, TLR4 is located on smooth muscle, epithelial cells and on immune cell types such as eosinophils and neutrophils [8,9], so has the potential to both directly and indirectly affect airway contraction and relaxation.

Lung slice preparations containing small intrapulmonary airways more closely resemble the morphology and functionality of the respiratory tract than isolated airways, and have been used to assess the influence of inflammation on reactivity [10,11]. A single study using mouse lung slices has shown that treatment with LPS for 24 h profoundly increased the expression and release of the pro-inflammatory cytokines and chemokines, TNFα, IL-1β, IL-5, IL-12, G-CSF, RANTES and eotaxin. [12]. Although *in vitro* treatment with LPS or some of these individual cytokines has been shown to alter tracheal responses to constrictor and dilator agents [9,13–16], the effects of LPS on small airway reactivity in physiologically relevant lung slice preparations remains to be determined.

LPS is commonly used to induce systemic and lung inflammation in mice and rats. Of relevance to the current study, intranasal administration of LPS to mice over four consecutive days induces *in vivo* airways hyperresponsiveness (AHR) to methacholine (MCh), mediated through the conventional MyD88-dependent signaling pathway and associated with increased neutrophils and total cell number in the BAL fluid [17]. Assessment of *in vitro* reactivity in lung slices from mice treated with LPS *in vivo* has the potential to define the influence of this LPS-induced inflammatory cell recruitment and cytokine production on small airways.

The aim of this study was to examine the effects of LPS on small airway reactivity using lung slices from naïve mice treated with LPS *in vitro* and lung slices from LPS-treated mice to test the hypothesis that LPS treatment would increase contraction and impair dilator responses in small airways. Airway contraction to MCh and serotonin (5-HT) and relaxation to β-adrenoceptor agonists and rosiglitazone (RGZ), a PPARγ agonist recently described as a novel bronchodilator [18], were assessed. Although LPS-induced inflammation was established both *in vitro* and *in vivo*, reactivity to all of the agents tested was not markedly altered. The experimental conditions that demonstrate AHR and impaired dilator responses in small airways in response to LPS remain to be defined.

## Methods

### Materials and solutions

Pentobarbitone sodium anaesthetic (Cenvet, Australia); ultra pure low melting point agarose, 10 x Hank’s Balanced Salt Solution (HBSS), 1 M HEPES buffer solution, Dulbecco’s Modified Eagle Medium (DMEM) (GIBCO/Invitrogen, Australia); rosiglitazone (Cayman); acetyl-β-methacholine chloride (MCh), isoprenaline hydrochloride (ISO), lipopolysaccharide (LPS), penicillin-streptomycin solution, salbutamol hemisulfate salt (SALB), serotonin hydrochloride (5HT) (Sigma-Aldrich, Australia).

LPS (Escherichia Coli serotype 026:B6: Sigma Aldrich Australia) stocks (1 mg/ml) were made up on day of use in either 1 x PBS for *in vivo* experiments or DMEM supplemented with penicillin-streptomycin for lung slice incubations.

### Ethics statement

All experiments were approved by the Animal Experimentation Ethics Committee of The University of Melbourne (application numbers 1011608, 1212485) and Murdoch Children’s Research Institute (MCRI, application numbers A738) and conducted in compliance with the guidelines of the National Health and Medical Research Council (NHMRC) of Australia.

### Animals

Specific pathogen-free male or female Balb/c mice (6–12 weeks) were obtained from the Animal Resource Centre Pty. Ltd. (Perth, Australia) or the Walter and Eliza Hall Institute for Medical Research respectively. Mice were housed at 20°C on a 12 h day/night cycle and fed a standard sterile diet of mouse chow with water allowed ad libitum.

### *In vivo* LPS administration

Female mice were anaesthetized by methoxyflurane (Medical Developments International Ltd, Australia) inhalation and 50 μl of PBS or LPS (5 μg of LPS in 50 μl of PBS) administered intranasally as previously described [19] at 9 am every day for 4 consecutive days.

### Lung slice preparation and *in vitro* LPS treatment

Lung slices were prepared as previously described [18,20,21]. Briefly, naïve male mice were culled by overdose of sodium pentobarbitone (0.4 ml) while LPS-treated female mice were euthanized with 0.4 ml of Avertin (2.5%) one day after final LPS administration. The trachea was cannulated (20 G Intima, BD Scientific) for inflation of the lungs by injecting approximately 1.4 ml of liquid agarose (44°C, 2% in HBSS supplemented with HEPES) followed by a bolus of air. After solidifying the agarose in 1 x HBSS/HEPES at 4°C for 20–30 min, the left lobe was isolated and mounted in a vibratome (VT 1000S, Leicamicrosystems) and bathed in 1 x HBSS/HEPES maintained at 4°C by surrounding the stage with ice.

Lung slices (150 μm) from all mice were serially sectioned and placed into a 24 well plate containing DMEM supplemented with 1% penicillin-streptomycin solution, 1 slice per well. Slices were then incubated in the absence or presence of 10 μg/ml LPS for up to 48 h. This concentration of LPS was used as an *in vitro* stimulus consistent with other related studies examining its inflammatory effects on mouse tracheal reactivity [8].

### Lung slice mounting and microscopy

Following incubation, slices were placed into HBSS/HEPES buffer prior to assessment of reactivity. Phase contrast microscopy was used to observe the lung slices on an inverted microscope (Diaphot 300; Nikon) using 10 x objective lens, zoom adaptor, reducing lens and camera (CCD camera model TM-62EX; Pulnix). Individual slices were placed on a 45 x 50 mm coverglass and covered in fine wire mesh (210 μm openings; Small Parts Inc.) with a small hole cut over an airway.

A custom-made perfusion chamber was created by running a channel of silicon along either side of the lung slice-nylon mesh preparation and covered with an additional 11 x 30 mm coverglass (resulting in approximately 100 μL volume within the channel). A single airway (200–400 μm) within each lung slice was selected for experimentation. Airway viability was established based on the presence of an intact layer of epithelial cells displaying ciliary activity and confirmation of reactivity to a constrictor agent.

**Fig 1:**
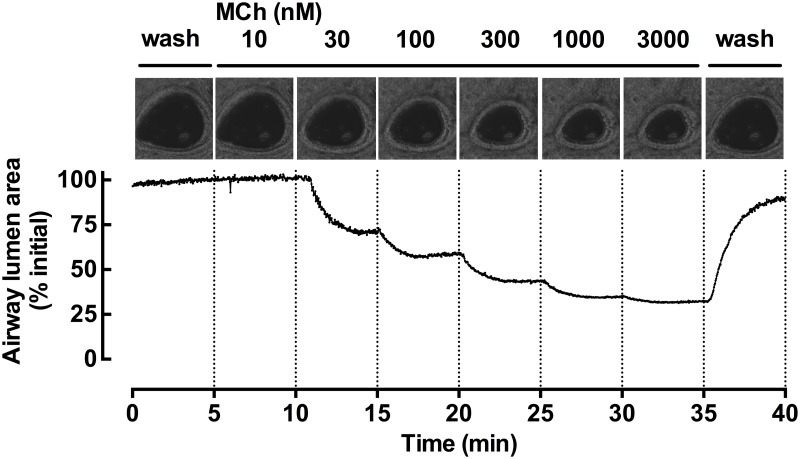
MCh contraction in mouse lung slices. Representative images of a single airway taken at the end of each 5 min perfusion with increasing concentrations of MCh. Representative trace of a MCh contraction recorded at 2 sec intervals as a % of the initial airway lumen area and shown over a total of 40 min perfusion.

Images showing decreases in airway lumen area in response to MCh and 5HT and relaxation of airways pre-contracted with 5HT in response to salbutamol, isoprenaline and rosiglitazone were recorded every 2 sec (0.5 Hz) using VideoSavant imaging software (VideoSavant; IO Industries, London, ON, Canada) and analysed using NIH/Scion (Scion Corp., Torrance, CA). A grey scale threshold distinguished between airway lumen and parenchyma for pixel counts. Representative images of a MCh contraction and area analysis are shown in Fig 1.

### Measurement of TNF-α, IL-10 and PGE_2_

Conditioned media from slices prepared from naïve mice was stored at -80°C. TNF-α and IL-10 were measured by ELISA according to the manufacturer’s instructions (R&D Systems, Minneapolis, Minnesota, USA). The ranges of detection were between 10.9–700 pg/ml and 15.6–1000 pg/ml for TNF-α and IL-10 respectively. PGE_2_ was measured by Enzyme Immunoassay (EIA) (Cayman Chemicals/Sapphire Bioscience). The range of detection of PGE_2_ was between 15–1000 pg/ml.

### Q-PCR

Lungs were harvested from PBS- and LPS-treated mice for Q-PCR analysis as previously described [22].

### Statistical analysis

All data were expressed as mean ± SEM, where each n represents one slice or lung lysate sample per mouse. Statistical analysis was performed using GraphPad Prism^TM^ (version 5.0c) with P<0.05 accepted as being statistically significant. For lung slice experiments, contraction data is presented as a % of the initial airway lumen area to correct for differences in airway size, while responses to dilators are expressed as % relaxation of pre-contracted airways. Concentration-response curves were fitted to obtain pEC_50_ and maximum values and analysed by unpaired t-tests or one-way ANOVA with Bonferroni’s *post hoc* test where appropriate. For TNFαELISA data and PGE_2_ EIA data were compared using non-parametric Wilcoxon matched pairs test. Q-PCR data was analysed using the ΔΔCt method compared to 18S as the control and analysed by Mann-Whitney non-parametric tests.

## Results

### Inflammatory effect of LPS treatment *in vitro*

**Fig 2:**
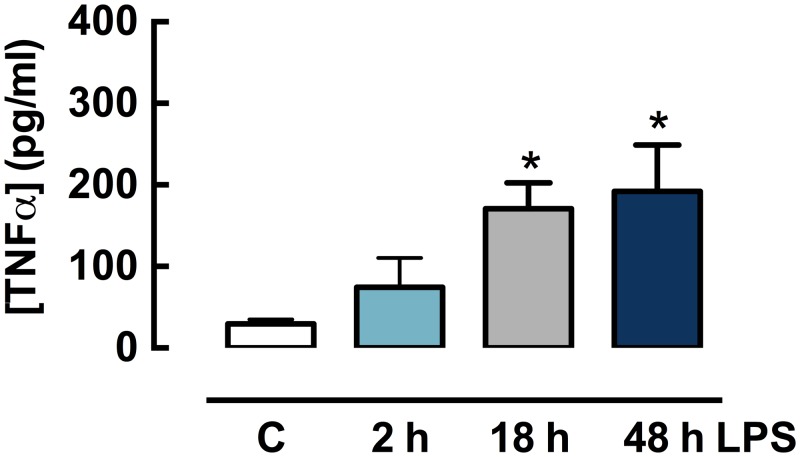
*In vitro* LPS treatment increases TNFα in conditioned media from mouse lung slices. Lung slices from naïve mice were cultured in the absence or presence of LPS (10 μg/ml) for 2, 18 or 48 h. Mouse TNFα levels were measured by ELISA (n = 6–20, where n represents conditioned media from one slice per mouse). Data is expressed as mean ± S.E.M. *P<0.05, non-parametric Wilcoxon matched pairs test, one tailed.

To assess whether LPS treatment was able to elicit an inflammatory response in lung slices, individual slices were incubated with LPS (10 μg/ml) for 2, 18 or 48 h. These timepoints were selected to observe potential LPS-induced changes during early and resolution phases of the inflammatory response, with a maximum of 48 h to minimise any effect due to loss of tissue viability. Mouse TNFα levels in conditioned media increased in a time-dependent manner (approximately 5-fold by 18 h, 6-fold by 48 h), confirming that LPS increases this pro-inflammatory cytokine (48 h: 175.6±65.9 pg/ml) (Fig 2).

### Effect of LPS treatment *in vitro* on bronchoconstrictor responses in lung slices

**Fig 3:**
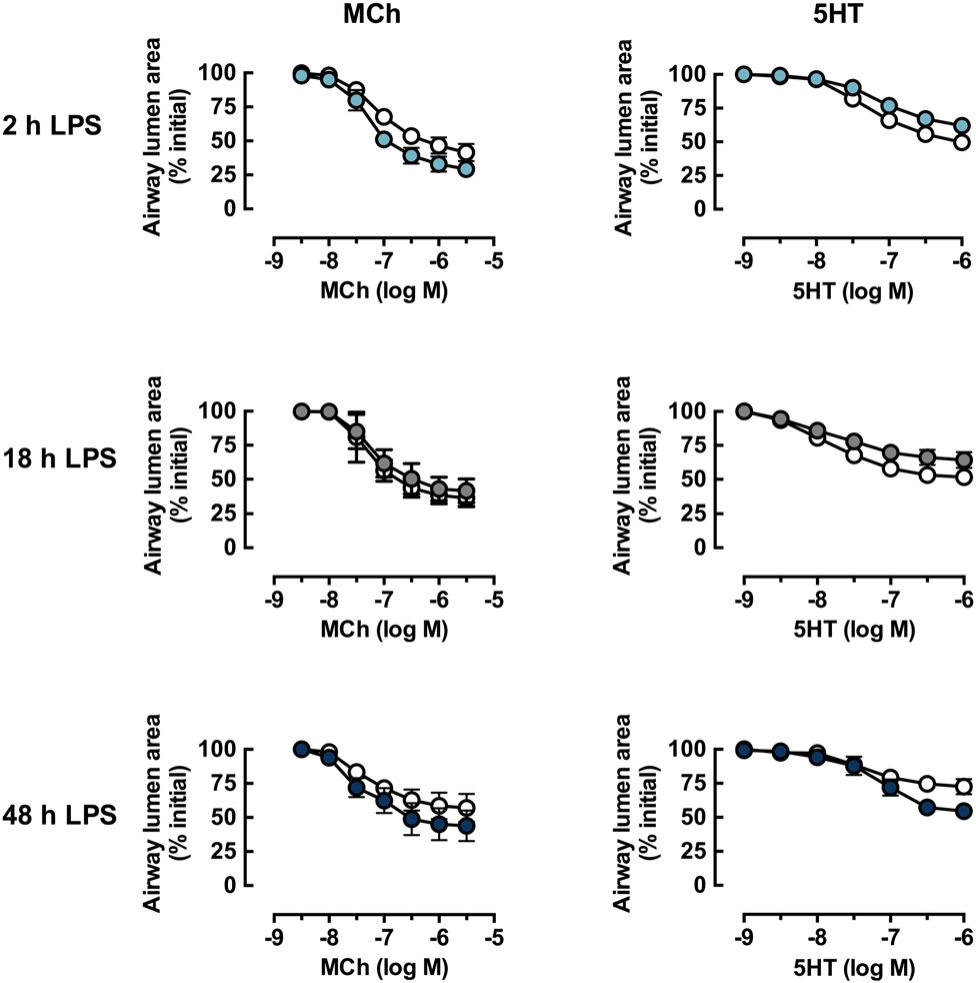
*In vitro* LPS treatment does not increase contraction to MCh and 5HT in mouse lung slices. Lung slices from naïve mice were cultured in the absence (open symbols) or presence (closed symbols) of LPS (10 μg/ml) for 2, 18 or 48 h for MCh (left hand side) or 5HT (right hand side) concentration-response curves. Average small airway contractile responses are expressed as % initial airway lumen area (mean ± S.E.M) for n = 3–4 where n represents paired slices from one mouse.

Having confirmed that *in vitro* exposure to LPS induced a marked inflammatory response, the direct effects of LPS on airway reactivity over the same time course were assessed. Slices were incubated with LPS (10 μg/ml) for up to 48 h, prior to perfusion with either MCh or 5HT (Fig 3). Both contractile agonists elicited concentration-dependent contraction with similar potency (Fig 3 and Table 1). Incubation with LPS did not affect MCh-induced contraction at any time-point (Fig 3 and Table 1). The potency of 5HT was not affected by LPS, but maximal 5HT-mediated contraction was slightly decreased at 2 and 18 h (P = 0.09, 0.03 respectively) and increased after 48 h (P = 0.11) (Fig 3 and Table 1).

**Table 1:** The effect of *in vitro* LPS treatment on potency and maximum contraction to MCh and 5HT.

	2 h	2 h	18 h	18 h	48 h	48 h
	Media	LPS	Media	LPS	Media	LPS
MCh pEC_50_	7.1±0.1	7.3±0.1	7.3±0.1	7.2±0.1	7.4±0.1	7.4±0.1
MCh maximum	59.6±6.4	72.3±5.1	66.4±3.4	60.6±5.6	43.5±10.1	57.0±12.2
5HT pEC_50_	7.2±0.1	7.1±0.2	7.9±0.03	7.8±0.07	7.3±0.2	7.1±0.2
5HT maximum	54.3±3.5	42.6±0.9	48.8±5.0	35.5±5.2*	29.3±5.7	52.2±5.5

Slices from naive mice were incubated in the absence or presence of LPS (10 μg/ml, 2–48 h). pEC_50_ and maxima were obtained from fitted individual concentration-response curves. Maximum represented as a % reduction in airway lumen area. n = 3–4 per group.

*P<0.05 *cf* media 18 h.

In addition to measurement of pro-inflammatory cytokines, IL-10 levels in conditioned media from LPS-treated slices were assayed. This was to determine if LPS could be releasing anti-inflammatory cytokines to oppose inflammation-induced changes in reactivity. Levels of IL-10 were not detectable under control conditions, and were not increased by up to 48 h incubation with LPS (n = 6–8, data not shown).

**Fig 4:**
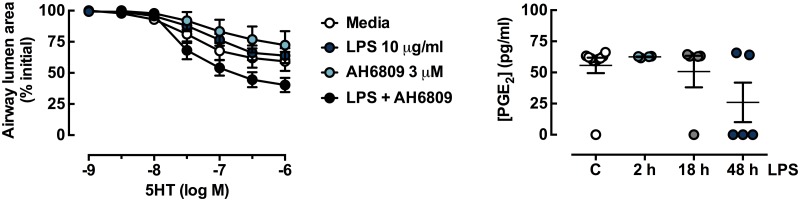
Contraction to 5HT is not altered by AH6809, EP1/2 receptor antagonist, and *in vitro* LPS treatment does not increase PGE_2_ in conditioned media from mouse lung slices. Lung slices from naïve mice were incubated for 48 h in the absence and presence of LPS (10 μg/ml) and/or AH6809 (3 μM) prior to 5HT concentration-response curves. Average small airway contractile responses are expressed as % initial airway lumen area (mean ± S.E.M) for n = 3–7 where n represents one slice per mouse. Lung slices were incubated in the absence and presence of LPS (10 μg/ml) for 2, 18 or 48 h. Conditioned media was stored at -80°C prior to PGE_2_ EIA. PGE_2_ levels (mean ± S.E.M) are for n = 4–10 where n represents conditioned media from one slice per mouse.

PGE_2_ release from the lung slices in response to LPS was also assayed, to determine if increased PGE_2_ could be opposing any cytokine-induced effects on reactivity. In addition, the effects of AH6809 (3 μM), an antagonist to the prostaglandin (EP) 1/2 receptors on airway smooth muscle, were assessed. These receptors have been previously shown to mediate relaxation to PGE_2_ in mouse lung slices [21]. There was no difference in concentration-response curves to 5HT in LPS-treated slices incubated in the absence or presence of AH6809 (Fig 4). In addition, LPS did not increase PGE_2_ release into the media of individual lung slices, as measured by PGE_2_ EIA (Fig 4).

### Effect of LPS treatment *in vitro* on bronchodilator responses in lung slices

**Fig 5:**
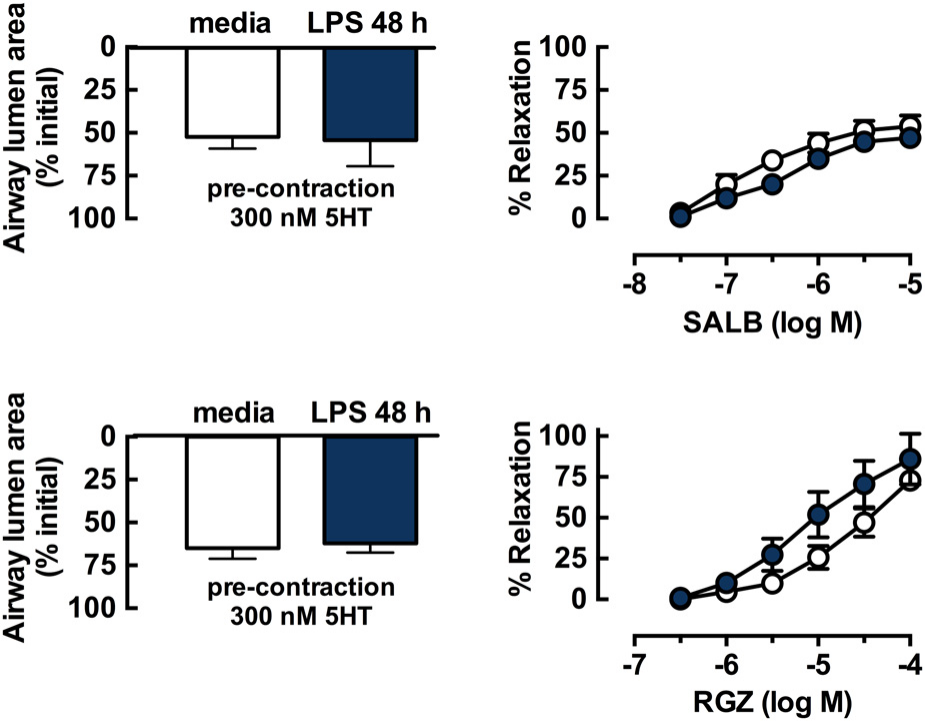
Partial relaxation to salbutamol or full relaxation to rosiglitazone in mouse lung slices is not impaired by *in vitro* LPS treatment. Lung slices from naïve mice were incubated for 48 h in the absence and presence of LPS (10 μg/ml). Small airways in mouse lung slices were pre-contracted with 300nM 5HT prior to perfusion with SALB (upper panel) or RGZ (lower panel). Average small airway contractile responses (left hand side) are expressed as % initial airway lumen area. Responses to SALB and RGZ (right hand side) are expressed as % relaxation of the submaximal pre-contraction. Responses (mean ± S.E.M) are shown for SALB (n = 3–4) and RGZ (n = 4–6) where n represents paired slices from one mouse.

To assess whether airway relaxation was impaired, despite the lack of marked changes in contractile responses to MCh and 5HT following LPS treatment, lung slices were pre-incubated with LPS for 48 h prior to assessment of dilator responses (Fig 5). The level of pre-contraction with 300 nM 5HT was similar in control and LPS-treated slices (~40% reduction in airway lumen area). SALB only caused partial relaxation at concentrations up to 10 μM (maximum relaxation, 52.1±14.9%), which was unaffected by treatment with LPS (Fig 5). RGZ elicited full relaxation but was less potent than SALB, requiring 100 μM to reverse the contraction to 5HT (RGZ pEC_50_: 4.4±0.2). Relaxation to RGZ was slightly increased following 48 h incubation with LPS (RGZ pEC_50_: 5.0±0.3, P = 0.13) (Fig 5). Incubation with LPS for only 2 h also had no effect on dilator responses to either SALB or RGZ (data not shown).

### Inflammatory effect of LPS treatment *in vivo*

**Fig 6:**
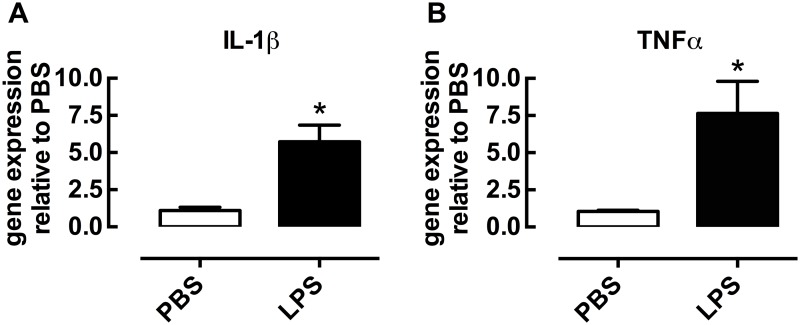
*In vivo* LPS treatment increases gene expression of pro-inflammatory cytokines in whole lung lysates. Mice were treated with PBS or LPS once a day intranasally for four days. On day 5, whole lungs from PBS- and LPS-treated mice were homogenized and RNA was extracted using a Qiagen kit. Following conversion of RNA to cDNA, Q-PCR was performed using Taqman primers. All data was analysed by the ddCt method, using 18S as the control gene. A) IL-1β expression and B) TNFα expression. All data is presented as gene expression relative to PBS (mean ±S.E.M). PBS n = 3, LPS n = 5 where n represents one lung lysate sample per mouse measured in triplicate. *P<0.05 Mann-Whitney non-parametric test compared to PBS.

Minimal effects on airway reactivity were observed following *in vitro* treatment of lung slices, despite evidence of increased inflammatory cytokine levels in conditioned media. To determine whether *in vivo* LPS treatment was required to alter reactivity, lung slices were prepared from mice treated with LPS for 4 days. To confirm that this *in vivo* LPS treatment regimen was able to elicit an inflammatory response in these mice, gene expression of the pro-inflammatory cytokines was measured in the lungs. IL-1β and TNFα were increased greater than 5-fold relative to PBS-treated controls (Fig 6A and 6B).

### Effect of LPS treatment *in vivo* and *ex vivo* on bronchoconstrictor and bronchodilator responses in lung slices

**Fig 7:**
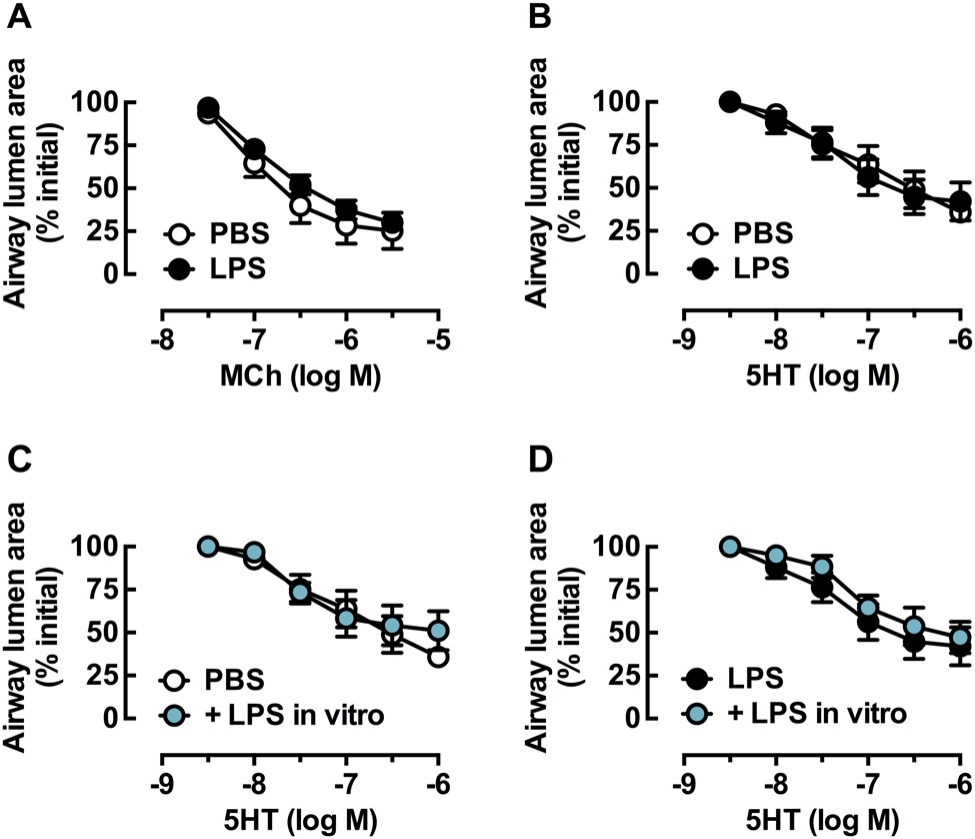
Constrictor responses to 5HT and MCh in lung slices from PBS and LPS-treated mice treated *ex vivo* with LPS. Mice were treated with PBS (open circles) or LPS (closed circles) once a day intranasally for four days. On day 5, lung slices were prepared and incubated in culture media overnight. Concentration-response curves were prepared to A) MCh and B) 5HT. Additional slices from PBS- and LPS-treated mice were incubated in media or LPS (10 μg/ml for 48 h, shown in blue). Concentration-response curves were prepared for 5HT C) PBS-treated mice ± LPS, D) LPS-treated mice ± LPS. All data is expressed as % initial airway lumen area (mean ± S.E.M), n = 3–4 where n represents one slice per mouse.

To assess the effects of LPS-induced lung inflammation *in vivo* on airway reactivity, lung slices were prepared following 4 day treatment with PBS or LPS *in vivo*. Contraction to MCh and 5HT was similar in lung slices from PBS- and LPS-treated mice, with no differences in potency or maximum reduction in lumen area (Fig 7A, 7B and Table 2). To determine whether sustained exposure to LPS *ex vivo* was required to influence reactivity, lung slices prepared from PBS- and LPS-treated mice were also treated with LPS (10 μg/ml) for 48 h. However, this did not affect contraction to either agonist (5HT, Fig 7C, 7D and Table 2; MCh Table 2).

**Table 2:** The effect of *in vivo* LPS treatment on potency and maximum contraction to MCh and 5HT.

	PBS	PBS	LPS *in vivo*	LPS *in vivo*
	Media	+ LPS *in vitro*	Media	+ LPS *in vitro*
MCh pEC_50_	7.2±0.2	7.6±0.1	7.0±0.1	6.7±1.6
MCh maximum	22.0±11.0	23.3±4.7	27.7±5.5	23.7±15.8
5HT pEC_50_	7.2±0.4	7.7±0.1	7.4±0.2	7.2±0.1
5HT maximum	22.1±8.9	48.9±12.9	38.3±10.4	42.2±11.4

Slices were obtained from PBS- and LPS-treated mice, and incubated in the absence or presence of LPS (10 μg/ml, 48 h). pEC_50_ and maxima were obtained from fitted individual concentration-response curves. Maximum represented as a % reduction in airway lumen area. n = 3–4 per group.

**Fig 8:**
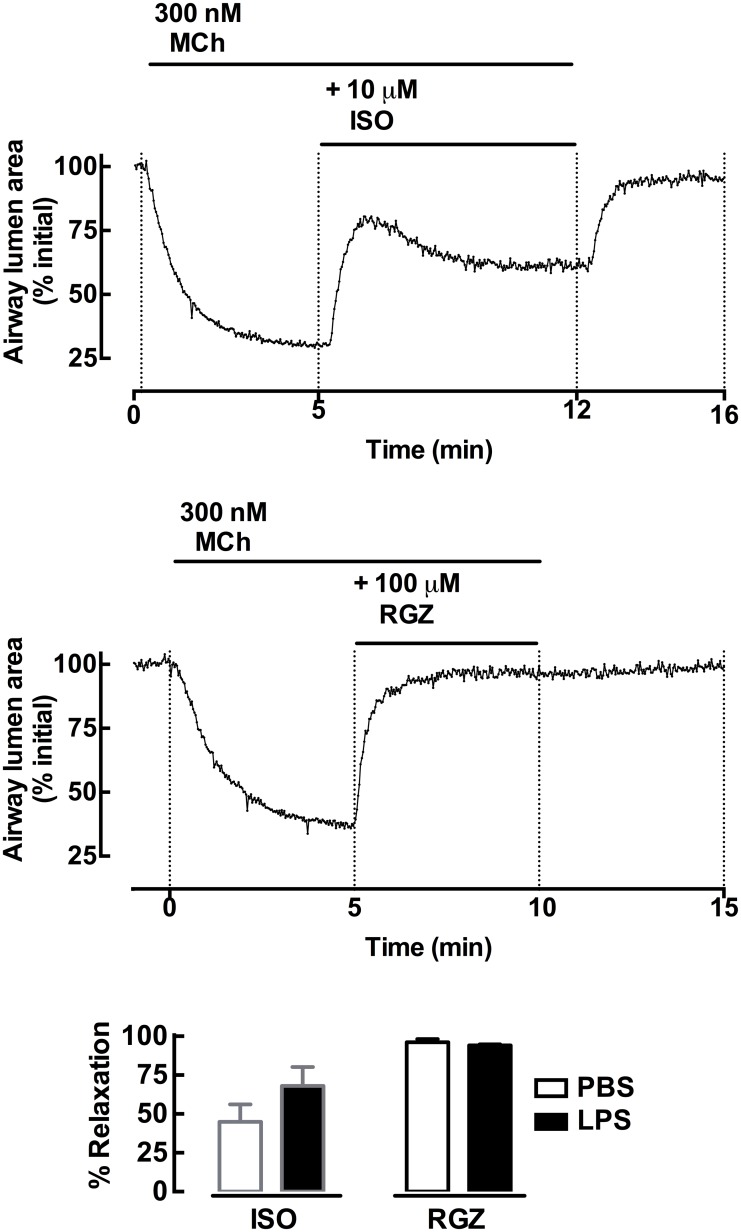
Bronchodilator responses in lung slices from mice treated with PBS or LPS for four days. Lung slices were prepared from PBS- (open bars) or LPS- (closed bars) treated mice. Airways were pre-contracted with 5HT (300 nM) prior to addition of ISO (10 μM, green) or RGZ (100 μM, blue). Upper panel: representative trace of the protocol of pre-contraction and relaxation to each dilator agent. Lower panel: grouped data of the bronchodilator responses are expressed as a % relaxation of the sub-maximal pre-contraction (mean ± S.E.M) for n = 3–4 where n represents one slice per mouse.

Bronchodilator responses were also assessed in lung slices from PBS- and LPS-treated mice. Airways were pre-contracted with 300 nM 5HT prior to addition of ISO (10 μM) or RGZ (100 μM), concentrations previously shown to be maximally effective in slices from naïve mice. There were no differences between the bronchodilator responses in the LPS-treated groups compared to the PBS-treated groups with either agent tested, with ISO causing partial relaxation and RGZ causing full relaxation (Fig 8).

### Effect of LPS treatment *in vivo* on expression of genes related to contraction

To assess the effect of LPS treatment *in vivo* on gene expression of receptors for MCh, 5HT, the G proteins coupled to these receptors and their downstream signaling receptors IP_3_R and RyR, whole lung lysates were prepared from mice treated with LPS *in vivo* for 4 days and PBS-treated controls. There was significant upregulation of the muscarinic M2 receptor, but there were no changes in the muscarinic M3 receptor, 5HT2A receptor, or their G proteins. There were also no changes in the IP_3_R isoforms and RyR isoform 1 and 3, however there was 5-fold upregulation of RyR isoform 2 (Table 3).

**Table 3:** The effect of *in vivo* LPS treatment on gene expression in whole lung lysates.

Gene	PBS	LPS
Muscarinic M3 receptor	1.04 ± 0.20	1.55 ± 0.32
5HT2A receptor	1.07 ± 0.26	1.06 ± 0.20
Gnai isoform 1	1.01 ± 0.11	1.22 ± 0.13
Gnai isoform 2	1.05 ± 0.24	0.69 ± 0.11
Gnai isoform 3	1.03 ± 0.18	0.87 ± 0.11
Gnaq	1.04 ± 0.20	1.18 ± 0.19
RyR isoform 1	1.12 ± 0.38	0.57 ± 0.08
RyR isoform 2	1.09 ± 0.31	4.96 ± 0.99 *
RyR isoform 3	1.01 ± 0.32	1.57 ± 0.35
IP3R isoform 1	1.01 ± 0.09	1.18 ± 0.10
IP3R isoform 2	1.00 ± 0.01	1.20 ± 0.18
IP3R isoform 3	1.02 ± 0.15	0.77 ± 0.11

Lung lysates were obtained from PBS- and LPS-treated mice for Q-PCR analysis using Taqman probes for M3 (chrm3) receptors, serotonin 2A receptors, G proteins, ryanodine receptors (RyR) and inositol trisphosphate (IP_3_) receptors. All data is expressed as mean ± SEM.

* P<0.05 unpaired t-test. PBS n = 3, LPS n = 5 per group.

## Discussion

This study characterised the potential influence of LPS-induced inflammation on small airway responses to constrictor and dilator agents by measuring changes in airway lumen area in lung slice preparations. Slices prepared from naïve mice were incubated with LPS for up to 48 h *in vitro* and slices were also obtained from mice treated with LPS daily for 4 days *in vivo*. Although LPS treatment *in vitro* caused the release of TNFα, an inflammatory cytokine known to contribute to AHR, changes in reactivity to constrictors were minimal. *In vivo* treatment with LPS elevated gene expression of pro-inflammatory cytokines. Despite this, there were no changes in the *in vitro* airway contraction in slices from these LPS-treated mice, even when they were also exposed to LPS *in vitro*. We report here for the first time that small airway responses to bronchodilators in mouse lung slices were similarly unaffected by LPS-induced inflammation.

Bacterial infections in patients with chronic lung diseases, such as asthma and COPD, can severely impact on their quality of life and lead to hospital admissions. We set out to assess whether exposure to LPS, a common bacterial toxin used to mimic bacterial infections in rodents, could cause inflammatory changes and alter airway reactivity in mouse small airways.

Initially, lung slices were incubated in the absence or presence of LPS *in vitro*. TLR4, the receptor for LPS, is present on airway smooth muscle and epithelial cells [8,9], and signaling through this receptor results in an up-regulation and release of multiple pro-inflammatory cytokines [23]. Here, we showed a time-dependent increase in TNFα release from lung slices following 2 and 18 h treatment with LPS, with a further modest increase up to 48 h.

We then assessed the potential influence of this LPS-induced inflammation, evidenced by the accumulation of TNFα on small airway reactivity. As previously reported, both MCh and 5HT contracted small airways in untreated lung slices [24]. However, changes in small airway reactivity to contractile agonists were not detected, irrespective of the levels of TNF03B1. This suggests that *in vitro* AHR can not be induced in mouse lung slices either during the initiation, progression or maintenance of the LPS-induced inflammatory response occurring over 48 h.

In untreated lung slices, the β_2_-adrenoceptor agonist SALB elicited only partial relaxation, while RGZ was less potent but able to fully reverse a submaximal 5HT-mediated contraction. RGZ has previously been shown to exert this acute dilator action via a PPARγ-independent mechanism involving inhibition of calcium oscillations and sensitivity [18,20]. LPS treatment *in vitro* did not affect dilator responses to either SALB or RGZ.

Previous experiments carried out in isolated mouse and rabbit trachea have demonstrated that LPS treatment *in vitro* can induce AHR to bradykinin and 5HT, but not carbachol as well as impair dilator responses to isoprenaline [25]. These agonist-specific effects are consistent with our findings for MCh, where LPS had no effect. The modest changes on the response to 5HT and salbutamol in the current study in comparison to those seen in trachea suggest that the effects of LPS may be less pronounced in smaller airways.

There are several other possible reasons for the lack of effect of LPS on reactivity in this setting. Although LPS treatment of lung slices has previously been shown to cause release of multiple pro-inflammatory cytokines known to induce *in vitro* hyperresponsiveness [23], their combined levels in the conditioned media may have been too low to increase contractile responses to MCh or have marked effects on the response to 5HT. In the current study, the accumulated levels of TNFα released from a single lung slice over 48 h were > 100 times lower than the TNFα concentration shown to increase contraction to acetylcholine in isolated human airways after 16 h exposure [14]. We have previously shown that 48 h incubation with 50 ng/ml TNFα had no effect on mouse airway contraction in lung slices [26], suggesting that mouse small airways may also be less sensitive to TNFα than human airways. Given the levels of TNFα detected in the current study, it is also unlikely that levels of other individual cytokines previously shown to be released in response to LPS [27], and demonstrated to induce *in vitro* AHR to MCh in mouse lung slices, such as IL-17A (100 ng/ml) [28], would have accumulated at sufficient levels to alter reactivity.

Alternatively, other endogenous mediators produced by lung slices in response to LPS may have opposed inflammation-induced changes in small airway reactivity. To address this possibility, we measured IL-10, a potent anti-inflammatory cytokine, in conditioned media collected after LPS treatment of lung slices *in vitro*. It is well documented that LPS can also increase levels of PGE_2_, a metabolite of the arachidonic acid pathway, which can inhibit inflammatory cytokine release from airway smooth muscle [29] and well as oppose contraction in small airways in mouse lung slices [21]. However, increased levels of IL-10 or PGE_2_ were not detected in the media following LPS treatment, possibly due to the limited production from the small volume of lung slice tissue relative to the conditioned media volume. Further investigation of the cytokine profile and their levels in conditioned media from lung slices following LPS treatment may reveal whether the overall production of cytokines from lung slices mediated through signalling via TLR4 is producing a balance of pro- and anti-inflammatory cytokines, minimizing potential changes in airway reactivity.

Given these findings, we do not believe that a lower concentration of LPS *in vitro* would have had altered either contraction or relaxation, since we attribute this lack of effect to either the difference in sensitivity of large and small airways to this inflammatory stimulus, or the minimal production of cytokines known to alter reactivity by lung slice preparations. Increasing the concentration of LPS *in vitro* to produce a greater pro-inflammatory response in lung slices may not be feasible, given that LPS can be toxic to cells. To provide a stronger stimulus, we assessed the effects of LPS given repeatedly and over a longer period *in vivo* prior to assessment of small airway reactivity *ex vivo*. We utilized a model in which 4-day exposure to LPS induces airway hyperresponsiveness, as measured *in vivo* using direct plethysmography [17]. In this more clinically relevant model, we hypothesized that LPS treatment *in vivo* could recruit inflammatory cells to the airways, to allow for subsequent release of pro-inflammatory cytokines from both inflammatory and resident cells, leading to changes in contraction and relaxation of small airways that would be maintained *in vitro*.

To validate the effects of *in vivo* LPS treatment, we initially assessed whether there were any overall changes in the expression of pro-inflammatory cytokines in total lung. As predicted, there were marked increases in gene expression for IL-1β and TNFα after LPS treatment, confirming an inflammatory response consistent with previous studies [19]. However, there were no LPS-induced changes in expression of the muscarinic M3 or 5HT_2A_ receptors that mediate contraction to MCh and 5-HT respectively. Similarly, the gene expression of receptors associated with downstream signaling for these contractile agonist receptors, namely IP_3_R isoforms 1–3 and RyR isoforms 1 and 3, were also similar in lungs from PBS- and LPS-treated mice.

Our results showed a 5-fold increase in the expression of RyR isoform 2 after LPS treatment *in vivo*. Based on previous studies, this isoform is more likely to be localized in vascular rather than airway smooth muscle in the lung [30]. It would therefore be of interest to conduct studies to assess the impact of this LPS-induced increase in RyR isoform 2 expression on vascular reactivity, also utiliising the lung slice method where these responses can be assessed [31].

Although contractile receptor expression was unchanged following LPS treatment *in vivo*, we assessed whether *in vitro* small airway reactivity had been altered in this model by other inflammatory mechanisms. We also treated slices from PBS- and LPS-treated mice with LPS to determine whether continued exposure to an inflammatory stimulus may be required to elicit or maintain changes in airway reactivity.

Given the evidence of *in vivo* hyperresponsiveness to MCh in this model [17], we predicted that small airway contraction to contractile agonists would be increased after LPS treatment *in vivo* and subsequent LPS incubation *in vitro*. However, we found no changes in the potency or maximum contraction either MCh or 5HT. While there were no changes in the bronchodilator efficacy of RGZ, there was a trend to increased relaxation to ISO at the single concentration tested after LPS treatment. This finding requires confirmation in full concentration-response curves, as ISO has very variable responses at single concentrations in mouse small airways [18,20].

We have previously reported on the differential effects of an inflammatory stimulus on large and small airway reactivity in the lung. In a chronic ovalbumin-induced model of allergic airways disease, *in vivo* hyperresponsiveness to MCh was evident in tracheal preparations *in vitro*, but not in small airways in lung slices [26]. However, these differential effects were attributed to the potential influence of small airway fibrosis and parenchymal interactions to oppose airway narrowing and inflammation-induced hyperresponsiveness in the distal lung. In the absence of remodeling changes with relatively short term LPS treatment in the current study, alternative mechanisms remain to be elucidated.

In summary, we have confirmed that treating lung slices with LPS can increase levels of TNFα, indicating that lung slices can be used as tools to assess immunomodulation of airway cells resident within functional tissue. We propose that the lack of effect of *in vitro* LPS treatment on small airway reactivity in this experimental setting may be due to the limited production of pro-inflammatory mediators to act in an autocrine fashion rather than increased levels of potential anti-inflammatory mediators. While treatment with LPS *in vivo* caused lung inflammation, as evidenced by increased expression of IL-1β and TNFα, there was no evidence of increased small airway contraction or impaired relaxation. The LPS-induced increase in RyR2 which predominates in the vascular smooth muscle warrants further investigation, as this may contribute to altered vascular reactivity in the inflamed lung. Despite causing inflammation, LPS failed to exert demonstrable effects on small airway reactivity within mouse lung slices. Alternative approaches are required to define the potential contribution of endotoxin-induced inflammation to impaired small airway function in inflammatory lung diseases such as asthma and COPD.
